# Progressive loss of PAX6, TBR2, NEUROD and TBR1 mRNA gradients correlates with translocation of EMX2 to the cortical plate during human cortical development

**DOI:** 10.1111/j.1460-9568.2008.06475.x

**Published:** 2008-10

**Authors:** Nadhim Bayatti, Subrot Sarma, Christopher Shaw, Janet A Eyre, Demetrius A Vouyiouklis, Susan Lindsay, Gavin J Clowry

**Affiliations:** 1 Institute of Neuroscience, Newcastle UniversityNewcastle-upon-Tyne, UK; 2 Institute of Human Genetics, Newcastle UniversityNewcastle-upon-Tyne, UK

**Keywords:** arealization, development, neurogenesis, subventricular zone

## Abstract

The transcription factors Emx2 and Pax6 are expressed in the proliferating zones of the developing rodent neocortex, and gradients of expression interact in specifying caudal and rostral identities. Pax6 is also involved in corticoneurogenesis, being expressed by radial glial progenitors that give rise to cells that also sequentially express Tbr2, NeuroD and Tbr1, genes temporally downstream of Pax6. In this study, using *in situ* hybridization, we analysed the expression of *EMX2*, *PAX6*, *TBR2*, *NEUROD* and *TBR1* mRNA in the developing human cortex between 8 and 12 postconceptional weeks (PCW). *EMX2* mRNA was expressed in the ventricular (VZ) and subventricular zones (SVZ), but also in the cortical plate, unlike in the rodent. However, gradients of expression were similar to that of the rodent at all ages studied. *PAX6* mRNA expression was limited to the VZ and SVZ. At 8 PCW, *PAX6* was highly expressed rostrally but less so caudally, as has been seen in the rodent, however this gradient disappeared early in corticogenesis, by 9 PCW. There was less restricted compartment-specific expression of *TBR2*, *NEUROD* and *TBR1* mRNA than in the rodent, where the gradients of expression were similar to that of *PAX6* prior to 9 PCW. The gradient disappeared for *TBR2* by 10 PCW, and for *NEUROD* and *TBR1* by 12 PCW. These data support recent reports that EMX2 but not PAX6 is more directly involved in arealization, highlighting that analysis of human development allows better spatio-temporal resolution than studies in rodents.

## Introduction

Cortical arealization controls the differentiation of the early embryonic cortical primordium, a neuroepithelial sheet that lacks any apparent regional-specific morphology or function, into the complex, regionally diverse mature cerebral cortex. Although stimuli arising from subcortical structures affect cortical differentiation, cortical-intrinsic influences drive the early phases of arealization. The genes controlling neocortical arealization are assumed to be expressed in graded or restricted patterns in order to be able to specify regional identities (Job & Tan, 2003; Mallamaci & Stoykova, 2006).

Two genes meeting these criteria in the rodent are *Emx2* and *Pax6* (O’Leary & Nakagawa, 2002). *Pax6* encodes a protein containing a paired domain and a homeodomain, and is mutated in patients with aniridia and in the mouse mutant small eye, *sey* (Ton *et al.*, 1991 1992), while *Emx2* is a homeobox-containing gene encoding a transcription factor that is one homologue of the anterior-head-specific *Drosophila empty spiracles* head gene (Simeone *et al.*, 1992). In mice, both *Pax6* and *Emx2* expression are found in the telencephalon during neurogenesis (E10.5–18.5; Walther & Gruss, 1991; Simeone *et al.*, 1992). In Pax6 *sey/sey* mutant mice, studies of the expression of areal markers such as *Cad6*, *Cad8* and *Id2*, have indicated a reduction in size of rostro-lateral areas and expansion of caudo-medial regions (Bishop *et al.*, 2000 2002). In Emx2 null mice, a reduction in size of caudal/medial cortical regions, together with an enlargement of those with rostral/lateral identity (Bishop *et al.*, 2000) is seen. Emx2 shows a complementary expression pattern to Pax6, and they downregulate each other in the cortex (Muzio *et al.*, 2002). Pax6 also controls glutamatergic neuronal cell fate in rodents (Kroll & O’Leary, 2005), and that the expression pattern of the T-box domain containing transcription factor *Tbr2*, a gene expressed by basal progenitors in the subventricular zone (SVZ), exhibits a similar high rostro-lateral, low caudo-medial expression pattern as that of *Pax6* (Bulfone *et al.*, 1999). Glutamatergic projection neurons and their progenitors sequentially express Pax6 followed by Tbr2 during development. This sequence of expression is followed by the basic helix-loop-helix, pro-neural gene NeuroD and the Tbr2-related layer VI marker, Tbr1 (Hevner *et al.*, 2006).

In the rodent, Emx2 and Pax6 are expressed in the ventricular zone (VZ) of the dorsal telencephalon that gives rise to cortical neurons, along two complementary tangential gradients. Although many mechanisms involved in rodent cortical development are shared in common with humans (Monuki & Walsh, 2001), the human cortex is composed of different and more complex local area identities that reflect differences in structure and function (Hill & Walsh, 2005). These differences need to be considered when extending findings from rodents to humans, and an important first step is to analyse expression patterns directly in humans. Therefore we analysed the temporal and spatial mRNA expression patterns of *EMX2* and *PAX6*, as well *TBR2*, *NEUROD* and *TBR1* from 8–12 postconceptional weeks (PCW). This relates to an early period of cortical development before innervation of thalamocortical fibres (Kostovic & Rakic, 1990; Meyer *et al.*, 2000).

## Materials and methods

All reagents were purchased from VWR International (Lutterworth, UK) unless otherwise stated.

### Human tissue

Brains were dissected from human foetal and embryonic terminations of pregnancies obtained from the MRC-Wellcome Trust Human Developmental Biology Resource at Newcastle University (HDBR, http://www.hdbr.org). Tissue from ages between 8 and 12 PCW (8 PCW, *n*= 5; 9 PCW, *n*= 3; 10–10.5 PCW, *n*= 3; 12–12.5 PCW, *n*= 3) were used with maternal consent and approval of the local University Hospital Ethical Review Committees. Age was estimated from measurements of foot length and heel to knee length. These were compared with a standard growth chart (Hern, 1984). Prior to sectioning, brains were fixed for 24 h at 4°C in phosphate-buffered saline (PBS) containing 4% paraformaldehyde (PFA), and then transferred to 70% ethanol for storage at 4°C. Samples were processed and then embedded in paraffin. Eight-micron-thick sections were cut, mounted on slides and used for *in situ* hybridization (ISH).

### Probe manufacture

For *PAX6* and *EMX2*, two sets of RNA probes were used yielding similar results. One set of RNA probes was used for each of *TBR1*, *NEUROD* and *TBR2*. In the case of *PAX6* and *EMX2*, sense and antisense probes were synthesized by transcribing linearized plasmid (pGEM3Z) containing 1200-bp (Emx2) and 720-bp (Pax6) fragments [nucleotides 730–1930 of GenBank accession no. NM_004098NM_004098 (Emx2) and nucleotides 416–1136 of GenBank accession no. BC011953 (Pax6)] with T7 or SP6 RNA polymerases. Additionally, a DNA template for polymerase chain reaction (PCR) was prepared for the production of the second set of probes from a reverse transcription first-strand synthesis reaction from RNA that had already been extracted from a 5-mm slice dissected from the caudal part of a human foetal cortex (9 PCW). PCR was carried out using primers (MWG, Ebersberg, Germany) specific for either EMX2 or PAX6 flanked by consensus sequences for T7 (anti-sense primer) and SP6 (sense primer) RNA polymerases. Probes for *TBR2*, *NEUROD* and *TBR1* were also manufactured in a similar manner by PCR from a cDNA template. The following primers were used (gene-specific sequence underlined): **EMX1 T7** AS: 5′-TAA GTT AAT ACG ACT CAC TAT AGG GCG AGT CAT TGG AGG TGA CAT CGA TGT CC;**EMX1 SP6** S: 5′-AAT ACG ATT TAG GTG ACA CTA TAG AAT ACC GCT GAC CGT GCA TCC GGC GCA C;**EMX2 T7AS**: 5′-TAA GTT AAT ACG ACT CAC TAT AGG GCG AGG CTG AGG CTG TGT GCC AGC TGC; **EMX2 SP6S**: AAT ACG ATT TAG GTG ACA CTA TAG AAT ACC AAG CGC TGC TTC ACC ATC GAG TC; **PAX6 T7AS**: TAA GTT AAT ACG ACT CAC TAT AGG GCG ATA GTG CAT GTT GTT CCA GGTT; **PAX6 SP6S**: AAT ACG ATT TAG GTG ACA CTA TAG AAT ACC TTC ACA TCT GGC TCC ATG TT;**TBR1 T7AS**: 5′-TAAGT TAA TAC GAC TCA CTA TAG GGC GA CAC CAT CTG CCC ATT GTT ATT TGA;**TBR1 SP6S**: 5′-AATACG ATT TAG GTG ACA CTA TAG AA TAC TAC CAA GGA GCT CCG TTC TAC CAG;**TBR2 T7AS**: 5′-TAAGT TAA TAC GAC TCA CTA TAG GGC GA CTA GTT TGT TGG TCC CAG GTT GCT;**TBR2 SP6S**: 5′-AATACG ATT TAG GTG ACA CTA TAG AA TAC AAT ACC AAC CCC GAC TGC ATA TTG;**NEUROD T7AS**: 5′-TAAGT TAA TAC GAC TCA CTA TAG GGC GA ATC TCC GAC AGA GCC CAG ATG TAG;**NEUROD SP6S**: 5′-AATACG ATT TAG GTG ACA CTA TAG AA TAC TGA CCA AAT CGT ACA GCG AGA GTG. The particular gene-specific regions were selected to ensure that standard PCR conditions could be used as follows: 40 cycles of 95°C 15 s, 65°C for 30 s and 72°C for 45 s. The PCR product was electrophoresed on a 1.5% agarose gel, and bands were cut out and purified with a gel extraction kit following manufacturers’ instructions (Qiagen, Hilden, Germany). Purified product was diluted in water (1: 100) and used as a template in a second round of PCR (with similar conditions). The PCR product was subjected to electrophoresis through a 1.5% agarose gel, bands were cut out and purified as before. Identity of the product was confirmed by sequencing. Seventy-five nanograms of purified PCR product or 1 μg of linearized plasmid served as a template for the labelling reaction. Digoxigenin (DIG)-labelled RNA probes were manufactured using a DIG RNA labelling kit according to manufacturers’ instructions (Roche, Lewes, UK). The labelled RNA was purified by centrifuging through ProbeQuant G-50 micro columns (Amersham Biosciences, Chalfont St Giles, UK). Labelling efficiency was determined with a dot blot with control labelled RNA (DIG labelling kit; Roche).

### ISH

ISH was carried out as previously described (Moorman *et al.*, 2001), with some modifications. Briefly, slides were de-waxed in xylene, gradually hydrated in decreasing ethanol concentrations before incubation for 8 min in proteinase K (20 μg/mL) at room temperature. Sections were fixed for 20 min in 4% PFA/PBS, washed in PBS, treated for 10 min in 0.1 m triethanolamine (Sigma-Aldrich, pH 8.0)/0.25% acetic anhydride/0.2% HCl, dehydrated in increasing concentrations of ethanol and air-dried by filtered air stream. Labelled probe (300 ng) was used per 100 μL Dig Easy Hyb Mix (Roche). Probe/Hyb Mix (200 μL) was used to cover each slide. Slides were incubated in a hybridization chamber overnight at 68°C, washed in 50% formamide/2× standard sodium citrate (SSC) for 20 min at 65°C, followed by four washes with decreasing SSC concentrations at 50°C (2, 2× SSC washes and 2, 0.2× SSC washes, the final at room temperature). After briefly washing in 0.1 m Tris (pH 7.6)/0.15 m NaCl (Buffer 1), and blocking 10% foetal calf serum (FCS; Invitrogen)/Buffer1 for 1 h, sections were incubated with anti-DIG antibody (Roche; diluted 1: 1000 in 2% FCS/Buffer 1) at 4°C overnight. The slides were washed in Buffer 1 for 6 × 30 min, and DIG antibody was visualized with NBT/BCIP solution (Roche; 20 μL/mL) in 0.1 m Tris (pH 9.5)/0.1 m NaCl (Buffer 2). Developing was stopped by rinsing slides in Buffer 2 then distilled H_2_O followed by 1%HCl/methanol and dH_2_O. Sections were mounted using Aquamount. Comparison of staining between sense and anti-sense probes was carried out to ensure specificity (see Supporting information, Fig. S1).

### Immunohistochemistry

Paraffin sections were de-waxed in two changes of xylene and re-hydrated in decreasing concentrations of ethanol in water. Sections were then treated with 3% hydrogen peroxidase (Sigma-Aldrich) for 10 min, and boiled in 10 mm citrate buffer before incubation with primary antibody [Pax6: diluted 1: 300; Covance, Princeton NJ, USA; Emx2: 1: 200; Sigma-Aldrich; in 0.3% PBS with 0.3% Triton X-100 (PBS-T) and 3% horse serum; Vector Laboratories, Peterborough, England]. Sections were incubated in a moist chamber at 4°C overnight, washed in PBS-T and incubated with a corresponding biotinylated secondary antibody (Vector Laboratories; 1: 300 in PBS-T) at 4°C for 2 h. After a further PBS-T wash, slides were incubated for 1 h with streptavidin-horseradish peroxidase (Vector Laboratories; 1: 200 in PBS-T). Antibody–epitope interactions were visualized with 0.05% diaminobenzidine/0.003% hydrogen peroxidase in PBS (Sigma-Aldrich) for 10–20 min. Sections were then dehydrated in increasing ethanol concentrations, cleared in xylene and mounted using histamount (Vector Laboratories).

## Results

The localization and gradient expression patterns of *EMX2* and *PAX6* mRNA were analysed by nonradioactive ISH (sense controls are shown in supporting Fig. S1). Furthermore, the localization and gradients of *TBR2*, *NEUROD* and *TBR1*, genes downstream of *PAX6*, which are thought to be important in the neurogenesis of cortical projection neurons, were also examined.

### Laminar expression of EMX2 and PAX6 during early human cortical development

**Fig. 1:**
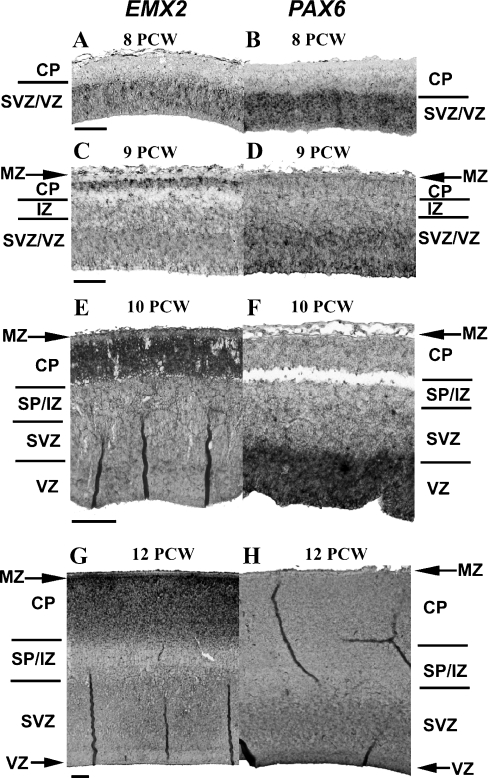
Laminar localization of *EMX2* and *PAX6* during early foetal development of the human neocortex. ISH revealed a changing laminar distribution of *EMX2* mRNA between 8 and 12 postconceptional weeks (PCW). Expression was observed only in the subventricular and ventricular zones (SVZ/VZ) at 8 PCW (A). However, by 9 PCW expression was also noted in the cortical plate (CP) (C). Subsequently, expression of *EMX2* intensified in the CP at 10 PCW (E) and 12 PCW (G), while still present in the SVZ and VZ at lower intensities. At 12 PCW, the highest level of *EMX2* mRNA expression was found in the CP most proximal to the marginal zone (MZ). *PAX6* was observed predominantly in the proliferative zones (SVZ/VZ) of the developing cortex (B, D, F, H). *PAX*6 mRNA was observed most intensely at 8 PCW in the VZ (A), after which a decrease was observed at 9 PCW (C). Staining intensity of *PAX6* decreased at 10 PCW and 12 PCW (F and H, respectively) due to the decrease in the relative thickness of the VZ. Sections for *EMX2* and *PAX6* are taken from the caudal and rostral poles respectively. Scale bars: 100 μm (A, C); 200 μm (E, G). IZ, intermediate zone; SP, subplate.

At 8 PCW, tissue ISH revealed expression of *EMX2* and PAX6 in the VZ and SVZ of the dorsal forebrain (Fig. 1A and B); however, by 9 PCW, expression of *EMX2* was also observed in the newly forming cortical plate (CP; Fig. 1C), where *EMX1* was also found to be expressed (in supporting Fig. S2), while *PAX6* remained in the proliferative layers (Fig. 1D). From 10 PCW, *EMX2* expression had switched to be predominantly within the CP, although lower levels were detected within the VZ/SVZ (Fig. 1E). By 12 PCW intense staining for *EMX2* mRNA was evident in the CP, particularly in the newly-forming outer layers, while showing a restricted pattern of distribution in the VZ (Fig. 1G). It is worth noting that EMX2 protein was generally found to localize in the same locations as the *EMX2* RNA (in supporting Fig. S3). *PAX6* RNA and protein expression was limited to the proliferative zones throughout all stages analysed (Fig. 1D,F and H; in supporting Fig. S3). Thus, *EMX2* showed clear differences in its expression between human (where it is detected in both the proliferating zones and in the CP) and mouse (where it is detected primarily in proliferating zones). In contrast, PAX6 expression follows a more similar pattern to that seen in rodents throughout development, being mainly localized to the proliferative layers.

### EMX2 and PAX6 gradients in the developing human cortex

**Fig. 2:**
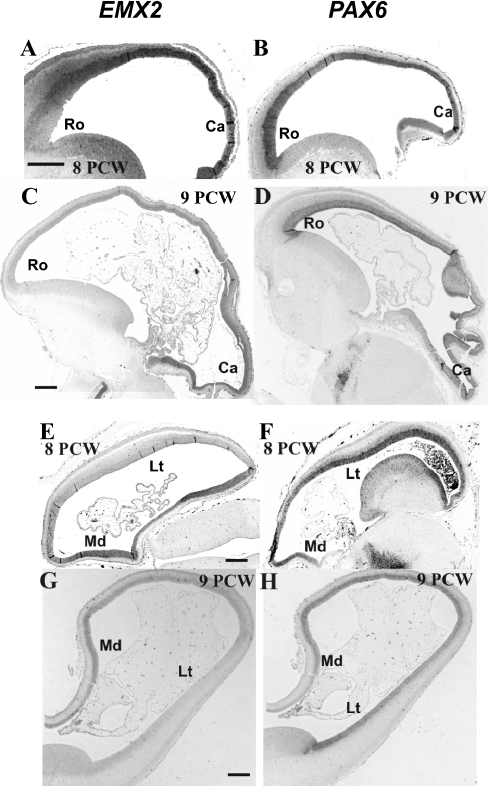
*EMX2* and *PAX6*gradients in the developing human cortex. ISH analysis of sagittal sections at 8 postconceptional weeks (PCW; A, B) revealed that *EMX2* and *PAX6* mRNA were expressed in reciprocal rostro-caudal gradients. While *EMX2* was expressed high caudally and low rostrally, *PAX6* showed an opposite gradient of expression. In sagittal sections at 9 PCW (C, D), *EMX2* mRNA maintained a similar caudal-rostral gradient to that seen at 8 PCW. The previously observed *PAX6* expression gradient, however, disappeared. Similarly, in horizontal sections at 8 PCW, *EMX2* and *PAX6* exhibited reciprocal opposing medial-lateral gradients (E, F). *EMX2* expression was observed high medially and low laterally with the opposite for *PAX6*. While the *EMX2* gradient still persisted at 9 PCW (G), the *PAX6* medial-gradient disappeared (H). Scale bars: 500 μm. Ca, caudal; Lt, lateral; Md, medial; Ro, rostral.

In rodents, *Pax6* and *Emx2* exhibit reciprocal and opposing caudal-rostral medial-lateral gradients that are instrumental in setting up cortical maps that control the expression of areal markers (Bishop *et al.*, 2000). Here we show that at 8 PCW both genes display similar gradients of expression in human (Fig. 2A,B,E and F).

At 8 PCW ISH for *PAX6* mRNA revealed a high rostral and lateral expression of the gene, with low caudal and medial expression (Fig. 2B and F). Conversely, *EMX2* showed a high caudo-medial to low rostro-lateral gradient in expression (Fig. 2A and E). The observed gradient of *PAX6* had disappeared by 9 PCW (Fig. 2D and H), although the *EMX2* gradient was maintained at 9 weeks (Fig. 2C and G) and at all other stages analysed (data not shown), even though *EMX2*-expressing cells had predominately translocated to the CP.

### Laminar expression of TBR2, NEUROD and TBR1 during early human cortical development

**Fig. 3:**
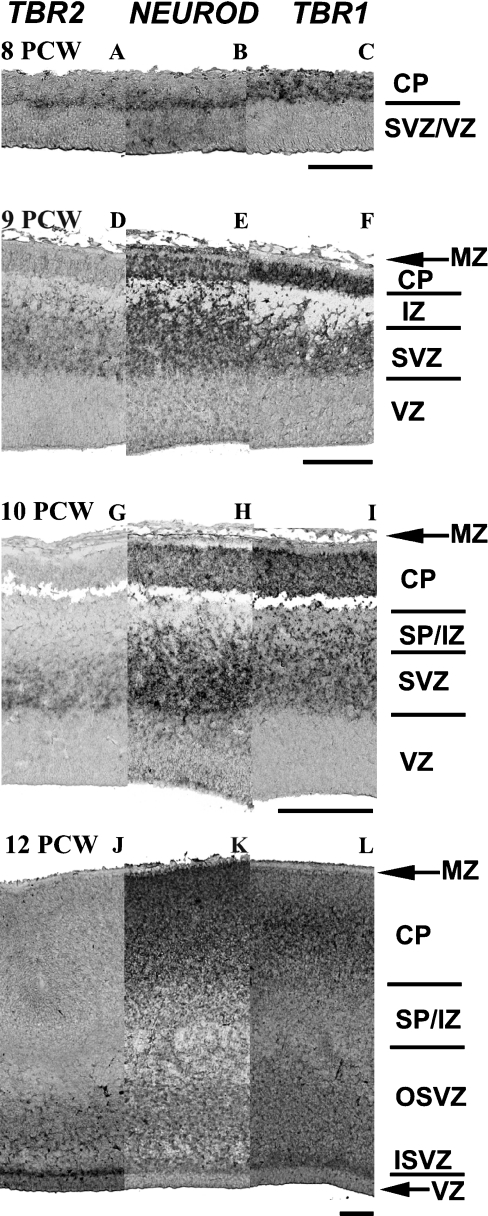
Laminar localization of *TBR2*, *NEUROD* and *TBR1* mRNA during early foetal development of the human neocortex. ISH analysis of *TBR2*, *NEUROD* and *TBR1* mRNA in the developing human cortex between 8 and 12 postconceptional weeks (PCW). At 8 PCW, *TBR2* and *NEUROD* exhibit restricted expression in what is probably the subventricular zone (SVZ) of the developing cortex (A, B), while *TBR1* expression additionally includes the cortical plate (CP; C). By 9 PCW, the SVZ is more distinctly identified at the border with the ventricular zone (VZ). All three genes are absent from the majority VZ but possibly present at the SVZ border, *TBR2* being restricted to the SVZ (D) while *NEUROD* and *TBR1* are additionally present in the CP (E, F). The expression patterns of these genes are similar at 10 PCW (G–I); however, *TBR2* exhibits a more restricted expression close to the SVZ/VZ border. By 12 PCW, all three show high expression in the ISVZ, while *NEUROD* and *TBR1* show more widespread expression in the SVZ and CP. Scale bars: 100 μm (C, F); 200 μm (I, L). ISVZ, inner SVZ; IZ, intermediate zone; MZ, marginal zone; OSVZ, outer SVZ; SP, subplate.

**Fig. 4:**
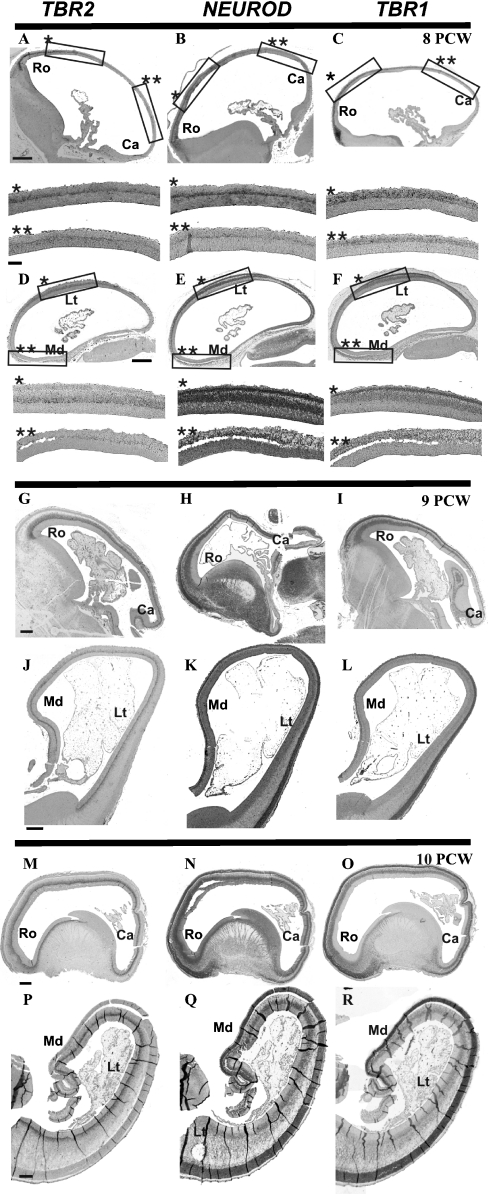
*TBR2*, *NEUROD*, and *TBR1* gradients in the developing human cortex. Expression analysis of *TBR2*, *NEUROD* and *TBR1* transcription factors temporally downstream of *PAX6* during neurogenesis demonstrates similar gradients of expression. At 8 postconceptional weeks (PCW), all three genes exhibited high rostral-low caudal (A–C), high lateral-low medial (D–F) expression. These gradients persisted at 9 PCW (G–I and J–L, respectively); however, the *TBR2* gradient was not as pronounced (G and J). By 10 PCW the *TBR2* gradient was absent (M and P), the *NEUROD and TBR1* gradients were becoming less pronounced (N, O, Q and R). By 12 PCW, there were no gradients of expression of any of these three transcription factors (data not shown). Higher magnification images of respective boxed areas are indicated by single and double asterisks. Sections shown are in the sagittal plane (A–C, G–I and M–O), horizontal plane (D–F) or coronal plane (J–L and P–R). Scale bars: 200 μm. Ca, caudal; Lt, lateral; Md, medial; Ro, rostral.

Tbr2, NeuroD and Tbr1 exhibit distinct compartmental-specific localizations in rodents (Hevner *et al.*, 2006). Here we have examined whether similar localizations are observed during the development of the human cortex between 8 and 12 PCW. At 8 PCW, *TBR2* RNA was located within a distinct layer between the VZ and the CP, probably corresponding to a newly formed SVZ. In a similar fashion, *NEUROD* was also found to be present in a discrete layer above the VZ, while *TBR1* exhibited a more widespread expression pattern including the SVZ and CP (Fig. 3A–C). It was interesting to note that *NEUROD* was expressed in the CP at 8 PCW in more lateral locations (see Fig. 4E). By 9 PCW, *TBR2* expression was predominantly limited to the SVZ, while both *NEUROD* and *TBR1* exhibited expression additionally in the CP, where most of the expression of *TBR1* was observed (Fig. 3D–F). These expression patterns were maintained for all three genes at 10 PCW (Fig. 3G–I). As the SVZ had expanded by 12 PCW, the majority of *TBR2* expression remained at the border of the SVZ and VZ, with less intense staining throughout the SVZ but most intense within the inner SVZ (Fig. 3J). *NEUROD* and *TBR1* were both mainly localized to the CP; however, both were expressed at the border of the SVZ and VZ and throughout the SVZ at lower levels (Fig. 3K and L). Taken together with the laminar localization of *PAX6*, these data show that although *TBR2*, *NEUROD* and *TBR1* exhibit some degree of compartmental-specific expression, together with *PAX6*, all four were expressed within the SVZ during early human corticogenesis, unlike in rodents where Tbr1 is absent (Hevner *et al.*, 2006).

### TBR2, NEUROD and TBR1 gradients in the developing human cortex

Tbr2, NeuroD and Tbr1 are sequentially expressed temporally downstream of Pax6 by progenitor cells undergoing differentiation and radial migration before finally residing in the CP as cortical glutamatergic projection neurons (Hevner *et al.*, 2006). Previous studies in rodents indicate that *Tbr2* is expressed in a high rostral-lateral, low caudal-medial gradient similar to *Pax6* (Bulfone *et al.*, 1999). At 8 PCW, *TBR2* mRNA, as well as *NEUROD*, and *TBR1* exhibited a gradient similar to *PAX6* (Fig. 4A–C, D–F). However, at 9 PCW, when the *PAX6* gradient has disappeared, the high rostral-lateral, low caudal-medial gradients of the other three transcription factors were maintained, although the *TBR2* gradient was less pronounced (Fig. 4G–I, J–L), and by 10 PCW it had definitely disappeared, and gradients for *NEUROD* and *TBR1* were barely discernible (Fig. 4M–O). By 12 PCW all gradients were absent (data not shown). Therefore the sequential loss of gradients of these transcription factors broadly mirrors the sequence in which these transcription factors are first expressed: *PAX6* at 9 PCW; *TBR2* at 10 PCW; and *NEUROD* and *TBR1* at 12 PCW.

## Discussion

**Fig. 5:**
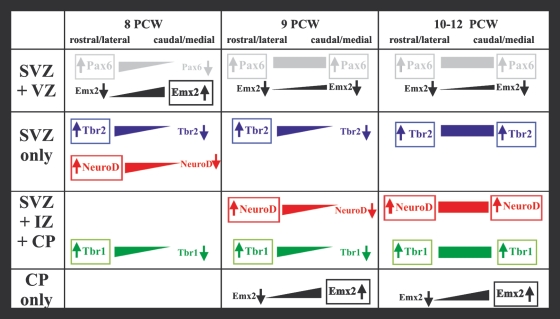
Summary of changes in expression patterns of *EMX2*, *PAX6*, *TBR2*, *NEUROD* and *TBR1* during early development of the human cerebral cortex. At 8 postconceptional weeks (PCW) *EMX2* and *PAX6* are localized within the proliferative zones of the developing cortex in opposing rostro-lateral/caudo-medial gradients. From 9 to 12 PCW onwards the majority of *EMX2* expression is found in the cortical plate (CP), where a similar gradient exists to that observed in the subventricular zone (SVZ)/ventricular zone (VZ). By this time the PAX6 gradient has disappeared and expression is widespread throughout the SVZ/VZ only. However, the mRNA of transcription factors downstream of PAX6 in neurogenesis form PAX6-like gradients in different compartments during this time period. *TBR2* exhibits a *PAX6*-like gradient within the SVZ until 10 PCW. *NEUROD*, which initially is not expressed in the CP, also forms a gradient within the SVZ at 8 PCW, which extends to the SP/intermediate zone (IZ) and CP at 9 PCW. The *TBR1* gradient is also observed from 8 PCW and encompasses all compartments outside of the VZ, most prominently within the CP, but disappears before 12 PCW. Thus, as *EMX2*-expressing cells migrate from the VZ to the CP, they pass through compartments expressing genes downstream of *PAX6* that exhibit *PAX6*-like gradients.

In order to examine the relative roles of *PAX6* and *EMX2* during arealization, we have examined the expression patterns of these two transcription factors using ISH during the early stages of corticogenesis in human. As we initially observed that the *PAX6* gradient disappeared early in corticogenesis, we also analysed the laminar localization and gradients of expression of *TBR2*, *NEUROD* and *TBR1*, genes temporally downstream of *PAX6* and expressed by cells during neurogenesis, in order to determine how they compare with *PAX6* (all gene expression patterns summarized in Fig. 5).

### Laminar localization of Emx2 and Pax6

Previous work in our laboratory has demonstrated the presence of *EMX2* mRNA in the proliferative zone of the developing human forebrain, at relatively low levels, from Carnegie stage 18 (CS18; 44 days; 6.5 PCW) until CS21 (52 days; 7.5 PCW; Lindsay *et al.*, 2005; Sarma *et al.*, 2005), when the CP starts to emerge (Meyer *et al.*, 2000). Here we report the expression of *EMX2* during the early stages of CP formation, where the functions of this gene are thought to be critical in modulating arealization mechanisms (Cecchi & Boncinelli, 2000). A principal finding of this study is that *EMX2* mRNA expression in the developing human cortex differs from that reported in rodents. In the rodent, *Emx2* mRNA localized chiefly to the VZ/SVZ (Cecchi, 2002), whereas in the human, expression of *EMX2* was present in the VZ/SVZ only during the early stages of CP formation; however, from 10 PCW onwards expression had switched predominantly to the CP. Emx2 has been reported to be expressed at low levels in the rodent CP, but only in the apical dendrites of CP neurons synapsing with the Cajal–Retzius cells of the marginal zone. In rodents, expression of *Emx1*, a transcription factor that is related to *Emx2*, is found to be expressed within the CP, but is not thought to be involved in mechanisms regulating cortical arealization (Gulisano *et al.*, 1996). However, double mutants indicate that Emx1 co-operates with Emx2 to regulate cortical size, lamination, neuronal differentiation, development of cortical efferents and thalamocortical path finding (Bishop *et al.*, 2003). Our observations that *EMX2* expression in the CP is reminiscent of that of *Emx1* in rodents lead to the intriguing possibility that *EMX1* in humans may take over *EMX2* function in cortical arealization in humans. However, the majority of *EMX1* expression is also found in the CP (in supporting Fig. S2) and this is consistent with previous reports in rodents.

We have previously demonstrated the early expression of the *PAX6* gene during human development, specifically within the dorsal forebrain at 6.5–7 PCW (Kerwin *et al.*, 2004; Lindsay *et al.*, 2005) by ISH, and from 8 to 12 PCW during early cortical development by immunohistochemistry (Bayatti *et al.*, 2008). We have extended these observations by analysing the extent and localization of *PAX6* mRNA expression during early human neocortical development. Unlike *EMX2*, the general laminar expression of *PAX6* in humans resembled that described in the rodent, being confined to the proliferative zones at all ages studied. The observation that *PAX6* and *EMX2* laminar-specific expression diverged as early as 9 PCW implies differing roles for these transcription factors during cortical development, whilst EMX2 and PAX6 may be important in regulating processes leading to the initial formation of the CP (at/or before 8 PCW; Gotz & Huttner, 2005). Additionally, PAX6 expressed in the human SVZ has been implicated in controlling the neurogenesis of γ-aminobutyric acid (GABA)ergic and glutamatergic neurons (Letinic *et al.*, 2002; Mo & Zecevic, 2008). Recently, analysis of a transgenic mouse model that carries several copies of the human *PAX6* locus, expressing up to three times more protein than the wild-type, provided evidence that PAX6 may not be directly involved in arealization. These mice exhibited no changes in Emx2 protein localization, and expression of the areal markers Id2 and Ephrin-B2 were also unaffected. However, reductions were seen in thickness of cortical layers, probably due to a reduced rate of proliferation in overexpressing cells (Manuel *et al.*, 2007).

A study identifying genes regulated by Emx2 in neuronal precursors *in vitro* has identified a gene profile that would be consistent with conferring maturation, i.e. differentiation of early precursors and/or induction of a migratory specification to these normally resident cells (Gangemi *et al.*, 2006). Given its expression gradient and location within the CP in humans, EMX2 may also be involved in the early stages of arealization and may control expression of areal markers directly. Recently, a study using nestin-Emx2 transgenic mice observed rostro-lateral shifts in the primary sensory and motor areas (Hamasaki *et al.*, 2004). As these mice do not show defects in thalamocortical pathfinding, regional-specific loss in cortical tissue or differences in *Fgf8* expression, which previously obscured interpretation of the function of Emx2 in Emx2 null mice, the authors concluded that Emx2 is directly involved in the mapping of primary cortical areas. Whilst our findings support these conclusions, we propose that in humans, due to its extended presence, EMX2 acts predominantly in the CP, in contrast to rodents where Emx2 in progenitors in the VZ is thought to impart positional identity to neuronal progeny.

### EMX2 and PAX6 gradients during early human cortical development

It has been proposed that Emx2 and Pax6 are two key regulatory genes that control neocortical arealization through expression in graded, restricted patterns in the embryonic cortex (O’Leary & Nakagawa, 2002; Job & Tan, 2003). This study demonstrates that in the developing human neocortex, *EMX2* and *PAX6* are expressed in counter caudomedial/rostrolateral gradients, in a similar manner to that observed in rodents. However, this pattern of expression is restricted to the period of development up to 9 PCW. Following this, *EMX2* maintains a gradient whereas *PAX6* becomes uniformly expressed throughout the proliferating zones of the cortex (Fig. 3D and H, respectively). If *EMX2* and *PAX6* play a role in cortical arealization in human development they are only able to interact up to the time the CP is initially formed and very soon thereafter.

PAX6 may therefore affect arealization indirectly. The first cells to populate the CP form layer VI and contribute cells to the subplate (Kostovic & Rakic, 1990). These cells are crucial in guiding thalamic afferents to different regions of the cortex, as they send out axons to the thalamus that meet incoming thalamic axons, providing guidance cues (Molnar & Blakemore, 1995; Hevner *et al.*, 2002). The regional identities of primary areas of the cortex are influenced by the thalamic innervation they eventually receive. Pax6 mutant mice exhibit defects in this thalamic innervation as well as in reciprocal cortical pathfinding (Hevner *et al.*, 2002), as axons do not reach their targets suggesting that Pax6 is important in the cortex for such events. As the first CP cells do not express Pax6, Pax6-dependent processes such as pathfinding may be mediated by other transcription factors such as Tbr1 and NeuroD that are expressed in differing compartments downstream of Pax6 during the process of progenitor migration and differentiation (Hevner *et al.*, 2006).

### Localization and gradients of PAX6, TBR2, NEUROD and TBR1

Although *PAX6* mRNA does not exhibit a gradient of expression from 9 PCW onwards in humans, it is possible that downstream transcription factors may be responsible for further arealization mechanisms in differentiating neurons migrating towards the pial surface. The compartmental localization of *TBR2* and *TBR1* mRNA is consistent with previous studies of the protein expression (Bayatti *et al.*, 2007). Our results further indicate that in addition to *TBR2*, *NEUROD* and *TBR1* form gradients similar to that of *PAX6* in compartments through which progenitors and subsequent immature neurons migrate during cortical development (summarized in Fig. 5). *TBR2* forms a gradient in the SVZ from 8 to 10 PCW, whilst *NEUROD* and *TBR1* also exhibit gradients in this compartment. Interestingly these gradients are also present in the CP for *TBR1* and *NEUROD* from 8 and 9 PCW, respectively, until at least 10 PCW. During this period TBR1, which is expressed by ‘early-born’ neurons including SP neurons and those in layer VI (Bulfone *et al.*, 1995; Hevner *et al.*, 2003), is expressed throughout the CP. Our previous observations show that the layer V marker Er81 appears later than 12 PCW in the human cortex (Bayatti *et al.*, 2007). All four of these transcription factors are localized within the SVZ, suggesting that PAX6–TBR2–NEUROD–TBR1 transitions all occur within this compartment. Considering the relative size and degree of differentiation of the human SVZ during development compared with that of the rodent, this observation supports a fundamental role for this compartment during corticogenesis. This study has highlighted that the location of *TBR2* is a particularly good marker for the SVZ at early stages. In particular it predominantly localizes to the inner subventricular zone (ISVZ) characterized in the macaque at E46, before the differentiation of the SVZ and appearance of an outer subventricular zone (OSVZ) (Smart *et al.*, 2002). The majority of *TBR2* expression is maintained in the ISVZ at later stages and corresponds to the junction between the ISVZ and VZ. Future experiments should characterize whether the SVZ contributes to arealization mechanisms, and if EMX2 and these transcription factors are expressed within the same cells during their migration and development. In addition, putative interactions between these transcription factors and EMX2 should be analysed, in a similar manner to that which PAX6 and EMX2 regulate each other in the VZ of rodents (Muzio *et al.*, 2002).

In conclusion, during human neocortical development, *EMX2* and *PAX6* expression patterns diverge at an early stage, when in rodents they are still presumed to be regulating the initial phases of arealization by reciprocal gradients of expression. However, *TBR2*, *NEUROD* and *TBR1*, temporally downstream genes of *PAX6*, maintain early PAX6-like gradients until 12 PCW in different compartments of the developing cortex (summarized in Fig. 5). The extended period of gestation in humans, as compared with that in rodents, enables finer spatiotemporal resolution of overlapping gene expression patterns. The majority of neurogenesis in the human forebrain occurs between 8 and 16 PCW (ten Donkelaar, 2000), in comparison to rodents in which similar events occurs within days. All the transcription factors described in this study are likely to possess additional functions during this extended period of human cortical development. Further analysis of the actions of these genes in humans using *in vitro* models may shed light on their functions during human development.

## Abbreviations

CP: cortical plate
CS: Carnegie stage
DIG: digoxigenin
FCS: foetal calf serum
ISH: *in situ* hybridization
PBS: phosphate-buffered saline
PBS-T: 0.3% PBS with 0.3% Triton X-100
PCR: polymerase chain reaction
PCW: postconceptional weeks
PFA: paraformaldehyde
SSC: standard sodium citrate
SVZ: subventricular zone
VZ: ventricular zone

## Supporting information

Fig. S1. Detection of anti sense and sense probes for *EMX1, EMX2, PAX6, TBR2, NEUROD and TBR1*.

Fig. S2. Laminar *EMX1* expression in the early human fetal cortex.

Fig. S3. Laminar localisation of EMX2 and PAX6 protein during early fetal development of the human neocortex.
