# Supplementary material for: Progressive loss of PAX6, TBR2, NEUROD and TBR1 mRNA gradients correlates with translocation of EMX2 to the cortical plate during human cortical development
Source: Eur J Neurosci. 2008 Oct;28(8):1449–56. doi: 10.1111/j.1460-9568.2008.06475.x (PMC2675014; doi:10.1111/j.1460-9568.2008.06475.x)
Supplement: Supplementary file 1 [file ejn0028-1449-SD1.doc]

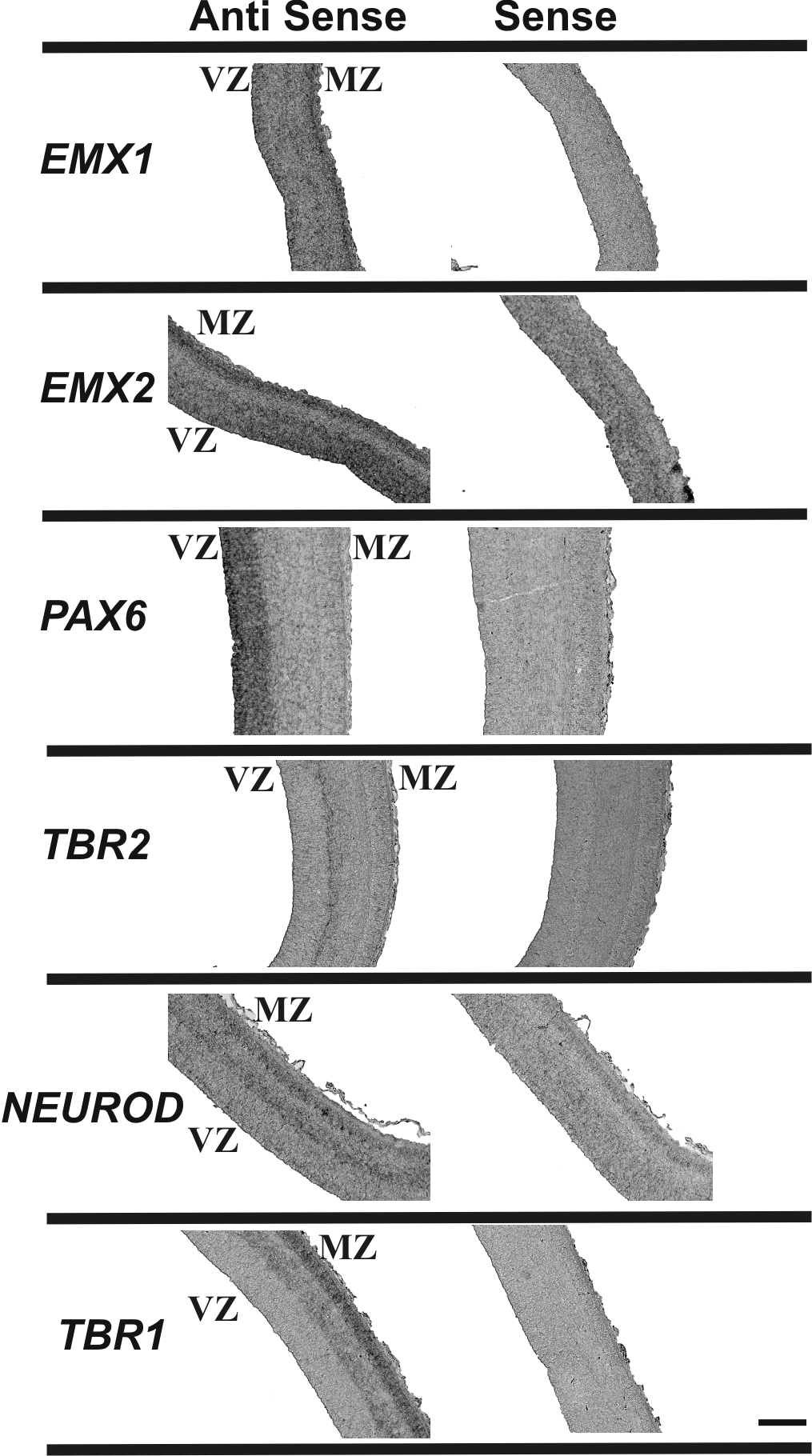


**Fig. S1**. Detection of anti sense and sense probes for *EMX1, EMX2, PAX6, TBR2, NEUROD and TBR1*. Specificity for the anti-sense probes was carried out in coronal sections (*EMX1* and *EMX2*, medial cortex, *PAX6*, *TBR2*, lateral cortex, *NEUROD, TBR1*, dorsal cortex) taken from 9 PCW. The marginal zone (MZ) and ventricular zone (VZ) are labeled for orientation purposes. Scale bar 200 µm.
