# Supplementary material for: Progressive loss of PAX6, TBR2, NEUROD and TBR1 mRNA gradients correlates with translocation of EMX2 to the cortical plate during human cortical development
Source: Eur J Neurosci. 2008 Oct;28(8):1449–56. doi: 10.1111/j.1460-9568.2008.06475.x (PMC2675014; doi:10.1111/j.1460-9568.2008.06475.x)
Supplement: Supplementary file 2 [file ejn0028-1449-SD2.doc]

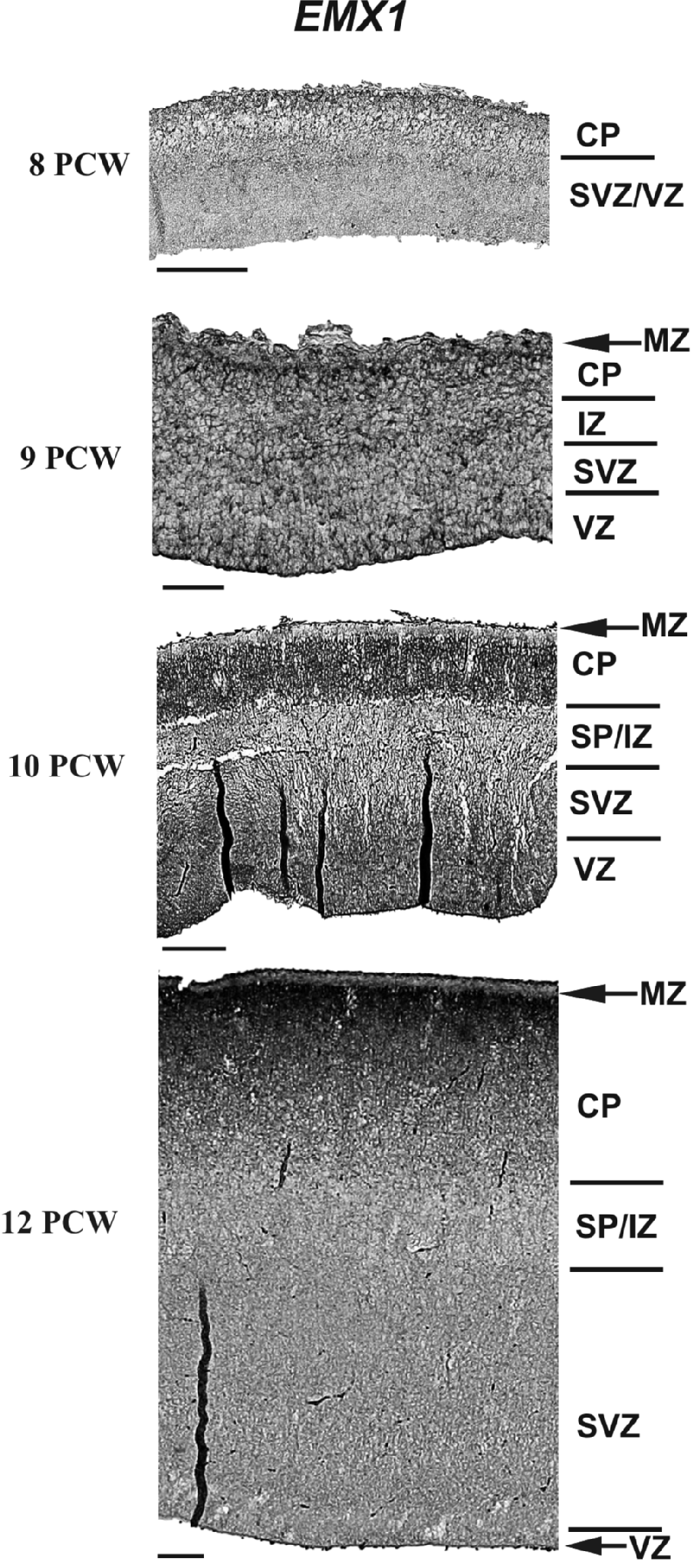


**Fig. S2**. Laminar *EMX1* expression in the early human fetal cortex. *In situ* hybridization revealed the highest level of *EMX1* mRNA expression in the outer parts of cortical plate most proximal to the marginal zone (MZ) at all stages studied. Some expression in the proliferative zones is also evident, particularly at 10 PCW (C). Scale bars 100 µm A, B, 200 µm C, D.
