# Supplementary material for: Progressive loss of PAX6, TBR2, NEUROD and TBR1 mRNA gradients correlates with translocation of EMX2 to the cortical plate during human cortical development
Source: Eur J Neurosci. 2008 Oct;28(8):1449–56. doi: 10.1111/j.1460-9568.2008.06475.x (PMC2675014; doi:10.1111/j.1460-9568.2008.06475.x)
Supplement: Supplementary file 3 [file ejn0028-1449-SD3.doc]

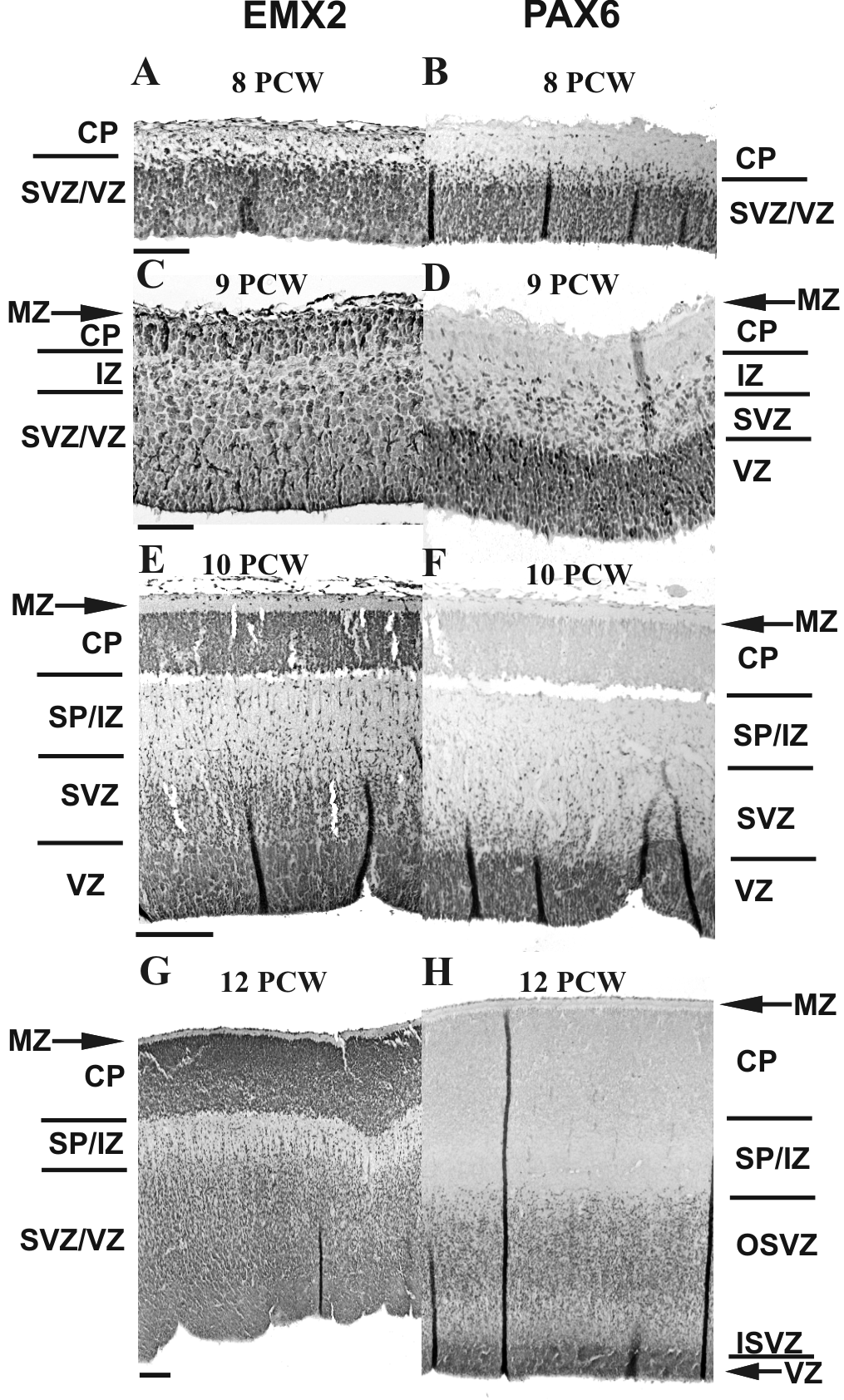


**Fig. S3**. Laminar localisation of EMX2 and PAX6 protein during early fetal development of the human neocortex. Immunohistochemistry revealed a changing laminar distribution of EMX2 protein between 8 and 12 PCW similar to that observed at the level of RNA. Expression was observed predominantly in the subventricular and ventricular zones (SVZ/VZ) at 8 PCW (A) and started to shift to the cortical plate (CP) by 9 PCW (C). EMX2 protein expression intensified in the CP at 10 PCW (E) and 12 PCW (G), while still present in the subventricular (SVZ) zone and VZ at lower intensities. PAX6 protein was observed predominantly in the proliferative zones (SVZ/VZ) of the developing cortex (B, D, F, H). Sections for EMX2 and PAX6 are taken from the caudal and rostral poles respectively. Scale bars: 100 µm (A, C), 200 µm (E, G). SP, Subplate; IZ, intermediate zone.
